# Uniting RNAi Technology and Conservation Biocontrol to Promote Global Food Security and Agrobiodiversity

**DOI:** 10.3389/fbioe.2022.871651

**Published:** 2022-04-25

**Authors:** Jonathan Willow, Samantha M. Cook, Eve Veromann, Guy Smagghe

**Affiliations:** ^1^ Chair of Plant Health, Estonian University of Life Sciences, Tartu, Estonia; ^2^ Laboratory of Agrozoology, Department of Plants and Crops, Faculty of Bioscience Engineering, Ghent University, Ghent, Belgium; ^3^ Biointeractions and Crop Protection Department, Rothamsted Research, Harpenden, United Kingdom

**Keywords:** RNA interference, dsRNA, transgenic crops, conservation biological control, integrated pest management, biosafety, sustainability, ecosystem services

## Abstract

Habitat loss and fragmentation, and the effects of pesticides, contribute to biodiversity losses and unsustainable food production. Given the United Nation’s (UN’s) declaration of this decade as the UN Decade on Ecosystem Restoration, we advocate combining conservation biocontrol-enhancing practices with the use of RNA interference (RNAi) pesticide technology, the latter demonstrating remarkable target-specificity *via* double-stranded (ds)RNA’s sequence-specific mode of action. This specificity makes dsRNA a biosafe candidate for integration into the global conservation initiative. Our interdisciplinary perspective conforms to the UN’s declaration, and is facilitated by the Earth BioGenome Project, an effort valuable to RNAi development given its utility in providing whole-genome sequences, allowing identification of genetic targets in crop pests, and potentially relevant sequences in non-target organisms. Interdisciplinary studies bringing together biocontrol-enhancing techniques and RNAi are needed, and should be examined for various crop‒pest systems to address this global problem.

## Global Biodiversity Initiatives in the Current Decade

Crops dominate about 11% of Earth’s land, and these areas are associated with rapid losses of species (Newbold et al., 2015), a trend that is expected to continue with an increasing human population (Zabel et al., 2019). Under the current trajectory of population growth on our planet, both sustainable agriculture and biodiversity conservation are becoming harder to achieve. International efforts have failed to slow down rapid losses in biodiversity, particularly urgent in the world’s tropical and subtropical regions, where agriculture continues to spread rapidly (Raven and Wagner, 2021). World leaders virtually gathered on 30 September 2020 at the United Nations (UN) Summit on Biodiversity, and many acknowledged that none of the Aichi Biodiversity Targets established in 2010 were met. Prior to the Summit, on 1 March 2019, the UN General Assembly proclaimed the decade of 2021–2030 as the UN Decade on Ecosystem Restoration, bringing hope that this conservation practice will mitigate a portion of biodiversity losses even when recent- and projected rates of biodiversity losses are alarming (Pimm et al., 2014; De Vos et al., 2015; Pimm and Raven, 2017; Ceballos et al., 2020). The UN’s declaration is also consistent with the creation of the Intergovernmental Science‒Policy Platform on Biodiversity and Ecosystem Services (IPBES), an independent body created to unify science and policy for promoting biodiversity, ecosystem services, well-being of human communities and sustainable development.

Another global initiative, the Earth BioGenome Project, aims to whole-genome sequence all extant eukaryotic species on Earth over the next decade, and provide open access to these genomes (Lewin et al., 2018). Given the estimated 1000 US dollars to draft-sequence an average vertebrate-sized genome, drafting a sequence for all known eukaryotic species is expected to cost 4.7 billion US dollars (Lewin et al., 2018). We are in an era where we have the resources to exploit advances in genomics for the benefit of species conservation on a global scale. Furthermore, there exist numerous Earth BioGenome Project-affiliated projects/initiatives involved in whole-genome sequencing; for example: 1000 Fungal Genomes (1KFG) Project, i5K (Sequencing Five Thousand Arthropod Genomes), Vertebrate Genomes Project (VGP), Bird 10,000 Genomes (B10K) Project, Darwin Tree of Life, BRIDGE Colombia, and African BioGebome Project (ABP). The strong list of projects/initiatives involved in obtaining whole-genomic data, which include global representation of many agriculturally relevant species, anticipates not only a prosperous outcome of this collective conservation initiative, but also its considerable applicability to sustainable crop production.

## Need for Integrating an Old Concept With New Technology

Agroecological communities are susceptible to changes in and around crops, likely due to multiple interacting stressors (Potts et al., 2010; Goulson et al., 2015), including extensive loss- and fragmentation of natural habitats, and agrochemicals applied for crop protection. Models of ecological resilience in agricultural landscapes predict associations between short-term benefits and long-term counterproductivity, but also the ability to mitigate negative trends through valuation of ecological services (Magnuszewski et al., 2015; Henderson et al., 2016). Progress toward sustainable food production and biodiversity conservation will undoubtedly require dramatic changes in attitudes and perceptions regarding policy, practice, and the adoption of new technologies.

Our aim here is to promote new ways to approach integrated pest management (IPM) research, a complex field that aims to identify holistic strategies that combine biological, physical and cultural tactics for optimizing the control of all classes of pests (i.e., invertebrates, pathogens, weeds, vertebrates), to achieve sustainable production of crops in a way that minimizes health- and environmental hazards and potentially reduces costs, through a decision-based process (Prokopy, 2003; Barzman et al., 2015; Wijnands et al., 2018; Creissen et al., 2021). IPM partially relies on conservation of biological control (biocontrol) agents relevant to pathogens and animal pests of a given crop and region. Biocontrol agents occurring in agroecosystems can ideally regulate pest populations to economically acceptable levels (Raaijmakers et al., 2009; Bender et al., 2016; Begg et al., 2017; Dainese et al., 2019). Measures for enhancing conservation biocontrol in crops can include preservation/restoration of adjacent natural and semi-natural habitats, maintaining crop diversity and landscape heterogeneity across spatiotemporal scales, and practicing inter-, under- and cover cropping locally. However, while local- and landscape-scale conservation measures can benefit biocontrol (Thies and Tscharntke, 1999; Li et al., 2014; Kovács et al., 2019), meta-analyses and models suggest that conserving natural and semi-natural habitats, either locally or at the landscape scale, results in variable success (Karp et al., 2018; Albrecht et al., 2020). Moreover, there are several reasons why these measures could fail to enhance biocontrol (Tscharntke et al., 2016; Begg et al., 2017). Thus, while conservation biocontrol measures comply with sustainability goals, these failures to enhance biocontrol services necessitate combined use with additional pest management techniques that are effective and biosafe.

Cautious use of pesticides is a pillar of IPM (Barzman et al., 2015). However, effects of pesticides on beneficial non-target organisms (Sgolastra et al., 2018; Simon-Delso et al., 2018; Meena et al., 2020; Schulz et al., 2021) supports the need to apply products that are target-specific. We advocate greater exploration into the use of double-stranded (ds)RNA due to its nucleotide sequence-specific mode of action that results in RNA interference (RNAi) after dsRNA uptake. In brief, when a sequence homology greater than 17 nucleotides in length exists between an endogenous, protein-encoding messenger (m)RNA and a small interfering (si)RNA fragment processed from dsRNA within the cell cytoplasm of the target species, the complementary region of endogenous mRNA can base-pair to the siRNA and become cleaved by the RNA-induced silencing complex (RISC) ribonucleoprotein (Kleter, 2020). This in turn prevents translation of the target mRNA; and the inhibition of protein synthesis results in the target phenotype (e.g., mortality, inhibition of reproduction, suppression of detoxification mechanisms). This sequence-specific mode of action makes dsRNAs the most target-specific pesticide compounds currently used in crop protection, in turn placing RNAi as potentially the most biosafe technique to combine with conservation biocontrol for sustainable IPM. RNAi-based crop protection technology can be employed using either *in planta* (i.e., transgenic) or exogenous (i.e., spray-based) delivery of dsRNA molecules to host-plant tissues (Taning et al., 2020).

As with any pesticide, the development of resistance must be considered; indeed, the potential for dsRNA resistance has recently been demonstrated in two herbivorous beetles, through selective breeding for this trait (Khajuria et al., 2018; Mishra et al., 2021). This represents a major hurdle for RNAi technology in crop protection (Khajuria et al., 2018; Yoon et al., 2018; Romeis and Widmer, 2020; Willow et al., 2021b; Mishra et al., 2021; Willow and Veromann, 2021; Christiaens et al., 2022; Darlington et al., 2022). Pesticide resistance management strategies that are already in use could potentially be adapted for RNAi. For example, strategies already in place for insect resistant (*Bt*) cotton and corn (Head and Greenplate, 2012), as well as midge-resistant wheat (Smith et al., 2004), in which susceptible plants are grown in refuge areas or as crop mixtures, could provide useful templates for resistance management of RNAi technology. The wheat–refuge strategy of Smith et al. (2004), in particular, demonstrated compatibility between conservation biocontrol and the use of a crop cultivar expressing a gene that confers antibiotic properties against a crop pest. Stacking genes in transgenic approaches represents another IPM option being pursued in order to reduce the risk of resistance-development, for example the dual expression of Cry3Bb1 *Bt* protein and an RNAi-inducing trait against corn rootworm (Levine et al., 2015). IPM options such as trap cropping (the use of attractive companion crops to divert pests from the cash crop) (Cook et al., 2007; Veromann et al., 2014; Sarkar et al., 2018) could also help slow resistance development by ensuring that not all members of a target population are affected by the pesticide. As with any other pesticide compound, dsRNA should be viewed as a reinforcement for pest suppression, with conservation biocontrol techniques representing the primary tactic for achieving sustainable protection of plants against pests. It is conceivable that this stronger promotion of conservation biocontrol, which could in turn encourage abundant populations of biocontrol agents and reduce reliance on RNAi-based control, holds the potential to prevent resistance development in some cases.

**FIGURE 1:**
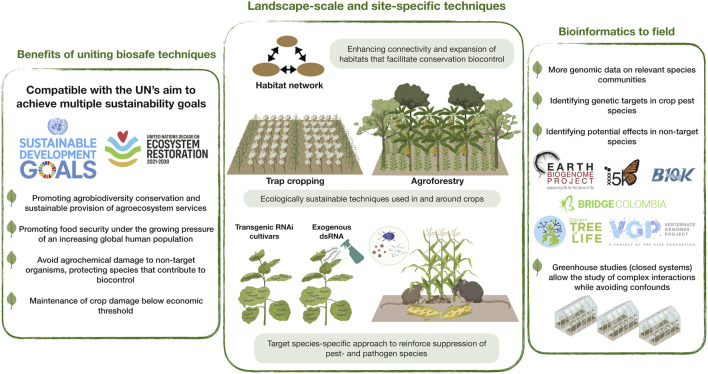
Conceptual scheme of uniting RNA interference (RNAi) and conservation biocontrol to sustain global food security and biodiversity. This addresses the United Nation’s (UN’s) sustainable development goals and the UN Decade on Ecosystem Restoration global initiative. This is enabled by landscape-scale connectivity of habitats, ecologically sustainable interventions in and around crops to support populations of biocontrol agents and biodiversity in general (e.g., trap cropping, agroforestry), and the use of species-specific RNAi approaches (e.g., RNAi cultivars, dsRNA spray). The latter will be supported by the generation of genomic/transcriptomic data on a wide range of eukaryotic species, including pathogens, animal pests and beneficial taxa (e.g., *via* Earth BioGenome Project and affiliated projects/initiatives). Studies should begin in lab and greenhouse, eventually to be scaled to field experiments.

Here we encourage an interdisciplinary approach for uniting conservation biocontrol- and RNAi techniques (Figure 1). This bridge promotes the long-held dual sustainability goal of achieving both global food security and biodiversity conservation, fitting the expectations of the UN Decade on Ecosystem Restoration. Our perspective also fits the ambitions of the Earth BioGenome Project, an effort valuable to RNAi-based pest control by enabling identification of specific genetic targets and ensuring an acceptable level of taxonomic specificity of dsRNA applications.

## RNAi: Market and Utility

Transgenic and spray approaches to RNAi-based crop protection have shown promise in targeting both insect pests and pathogens (Baum et al., 2007; Mao et al., 2007; Koch et al., 2016; Head et al., 2017; Mitter et al., 2017; Worrall et al., 2019; Kuo and Falk, 2020; Petek et al., 2020). Many transgenic approaches to crop improvement, including RNAi, could play a role in the diversification of IPM for agricultural sustainability (Anderson et al., 2019). RNAi cultivars, and all other transgenic crops, fall within the definition of “living modified organisms” according to the UN’s Convention on Biological Diversity under its Cartagena Protocol on Biosafety for transboundary movement. While current restrictions prevent field cultivation of transgenic RNAi cultivars in European Union (EU) countries, there is growing advocacy from RNAi experts for EU policymakers to re-think legislation and adapt to sustainability needs (Mezzetti et al., 2020; Taning et al., 2020; De Schutter et al., 2022). A well-known implementation of transgenic RNAi-based crop protection is the bioengineering and cultivation of RNAi papaya in Hawaii, whereby a transgene was introduced into the papaya genome for control of papaya ringspot virus. This cultivar rescued the Hawaiian papaya industry and currently dominates their market (Kuo and Falk, 2020). Another well-known example of RNAi-based crop protection is the development of RNAi maize by Monsanto (Baum et al., 2007; Head et al., 2017), prior to their acquisition by Bayer. Bayer has licensed this cultivar to other seed companies, in preparation for a 2022 launch in the US, followed by Canada in 2023.

Spray formulations, while not yet registered in any country, are currently under development by pioneering companies like Nufarm (Australia), GreenLight Biosciences (United States), RNAissance (United States) and Syngenta (Switzerland). Mitter et al. (2017) demonstrated that a single spray of dsRNA-loaded, biodegradable, non-toxic, layered double hydroxide clay nanosheets (collectively termed BioClay) can effectively protect Cowpea and tobacco from cucumber mosaic virus and pepper mild mottle virus for at least 20 days; this protection was observed on both sprayed- and newly-emerged unsprayed leaves. Petek et al. (2020) conducted a small field trial demonstrating control of Colorado potato beetle by spraying potato plants with a simple water-based preparation containing dsRNA. Transdermal delivery of dsRNA *via* topical exposure, resulting in RNAi, has also been observed in laboratory studies with aphids (Niu et al., 2019; Zheng et al., 2019; Yan et al., 2020a). The use of dsRNA sprays is a particularly interesting approach, as sprays may be altered with respect to co-formulants and/or target gene, in accordance with adaptive management needs. While spray-based management may require successive dsRNA applications within a single season (a potential confliction point for farmers), a recent study indicates that reduced dsRNA concentrations can be applied- and consumed over longer periods to achieve a similar RNAi-induced mortality rate compared to short-term consumption of higher dsRNA concentrations (Willow et al., 2021a). On the other hand, applying reduced concentrations may fail to eliminate target populations, thereby increasing the chance of resistance development. While these factors have important implications for optimal practice and economics of dsRNA spray regimes, the best approach will always be case-dependent, and several factors including crop, pest and formulation are key to developing the most economic and effective strategy.

Of notable utility to increasing cost-effectiveness of dsRNA production is the use of cell-free systems, as well as the use of dsRNase-deficient bacteria strains engineered to produce dsRNA-expressing genes. The latter can be cultured on growth media to produce large volumes of dsRNA-expressing bacteria, which can then be applied to plants as heat-killed bacteria, or as lysate after using a lysis buffer, or even as live cells. Whitten et al. (2016) demonstrated that genetically-engineered gut symbiont bacteria, of both a trypanosome-transmitting assassin bug and western flower thrips (a crop pest), were highly persistent in the insect colonies, resulting in sustained RNAi. Furthermore, engineered bacteria remained detectable in hosts for up to 250 days, being horizontally transmissible *via* inadvertent coprophagy. This potentially useful application of RNAi in crop protection requires further investigation into its utility for various crop‒pest systems.

A notable area of interest for both food security and biodiversity conservation is the potential application of RNAi in vertebrate pest management (reviewed by Horak, 2020). For example, islands can suffer considerable biodiversity losses due to invasive rodents, and dsRNA-based rodenticides represent a potential advancement in conservation efforts. Rodent outbreaks damage, on average, 10%–30% of food crops annually (John, 2014). Mitigation of these losses often depends on the use of synthetic chemicals and toxic baits, but these methods are criticized due to poor efficacy, risks posed to non-target species, and increased chemical load in the environment (Horak, 2020). Furthermore, some vertebrates (e.g., insectivorous birds and bats) contribute to biocontrol of crop pests, and thus need protection from broad-spectrum pesticides. The use of RNAi in targeting invasive vertebrate pests can mitigate losses of insectivorous vertebrates, thereby promoting their biocontrol services. The potential costs saved on safeguarding non-target island vertebrate communities, which contain some of the world’s most endangered species, may represent another economic benefit of RNAi technology, further illustrating the need to develop dsRNA-based rodenticides in place of anticoagulant rodenticides that present great risk to non-target vertebrates, including humans (Horak, 2020). While studies suggest barriers to dsRNA uptake in mice (Kleter, 2020), great strides in uptake are being made in insects and fungi (Wytinck et al., 2020), suggesting potential for enhancing dsRNA uptake in vertebrates. However, dsRNA fragments greater than 30 base pairs in length can activate an immunomodulatory response in vertebrates *via* the release of type I interferons (IFNs, a type of cell signaling protein for defense against viruses) (Whitehead et al., 2011). This can limit RNAi efficacy in vertebrates, and currently represents a major hurdle to progress in RNAi-based control of vertebrate pests, as does risk assessment which must be especially well considered before use on vertebrates proceeds on any scale.

## Ensuring Biosafety of dsRNA in Non-Target Organisms

RNAi’s role in protecting biodiversity lies in the environmental safety associated with both transgenic and spray approaches. While risk assessment of dsRNA is still under conceptual development (Arpaia et al., 2020; Rodrigues and Petrick, 2020; Romeis and Widmer, 2020), results of dsRNA risk assessments that have been conducted (Bachman et al., 2013; Pampolini and Rieske, 2020; Castellanos et al., 2022; Hollowell and Rieske, 2022) suggest that off-target gene silencing in a desirably-narrow range of species may represent the worst-case scenario. However, potential effects of dsRNA on non-target organisms are difficult to predict, since relatively few species have had their genomes and/or transcriptomes characterized. Also, the risk of activating immune IFN responses in vertebrates (which can induce apoptosis, a form of programmed cell death in multicellular organisms), as well as nonsequence-specific immune responses in some invertebrates such as the honey bee *Apis mellifera* (Flenniken and Andino, 2013), represents a serious factor to consider in risk assessment on non-target organisms. Furthermore, there are several routes of exposure to be considered, including direct consumption of dsRNA-contaminated plant tissues, topical contact with dsRNA, and trophic exposure to dsRNA (i.e., predation on dsRNA-contaminated herbivores, and exposure to dsRNA *via* other consequent food web associations). The hopeful outcome of acquiring whole-sequence information on a great diversity of species will be our ability to safeguard an unprecedented diversity of species in conservation initiatives; this high-resolution evidence of biosafety may prove to be reliably generated using bioinformatics tools (e.g., BLAST, OFFinder). Choosing appropriate species for RNAi risk assessment depends on the crop species of interest and the trophic interactions existing in and around the focal agroecosystem.

The scarcity of high quality open access sequences currently impedes our ability to safeguard non-target organisms with certainty. However, it is critical to exploit currently available transcriptomes, representing unique taxonomic lineages, as templates for designing species-specific dsRNAs; and subsequently examine RNAi susceptibility across these taxa. In both insects and fungi, dsRNA uptake and RNAi efficiency can vary between- and within taxonomic groups (Wytinck et al., 2020; Willow and Veromann, 2021), and both uptake and efficiency can change with formulation (e.g., nanoparticles) (Yan et al., 2020b; Wytinck et al., 2020). Intracellular, intercellular and intertissue transport of dsRNA should be a primary focus of investigations, enhancing our understanding of how dsRNA is trafficked within different groups of organisms. Experimentally examining RNAi susceptibility across a broad array of taxa will represent an avenue of great interest for both pest management and biodiversity conservation, in light of the growing development of RNAi technology for crop protection. While non-target tests that are conducted to support environmental risk assessments typically focus on taxa that provide important ecosystem services (e.g., pollinators, biocontrol agents, decomposers), other taxa that contribute to biodiversity and stability of agroecosystems should not be neglected. For example, many herbivorous insects within crops are of negligible- to no threat to crop yield, and furthermore represent important food sources for farmland vertebrates and invertebrates (Müller, 2018). Besides broadening the test-species selection for assessing environmental risk of pesticide technology (including that of RNAi), other adjustments and standardized procedures for RNAi risk assessments are needed. While dsRNA is perceived as low-risk, dsRNA represents a unique class of pesticide, and applications could potentially result in immunomodulatory effects in certain non-target organisms (Flenniken and Andino, 2013), as well as other effects currently unknown. RNAi environmental risk assessment should be conducted not only with the active ingredient (dsRNA), but also with the potential- or final market product, as alterations to formulation can affect both environmental persistence- and *in vivo* metabolism of dsRNA. For reliable confirmation of RNAi risk assessment results, ring testing of potential- or final market products should be conducted when possible, due to potentially high variability between research laboratories, as well as between non-target species populations.

The environmental fate of dsRNA presents an additional advantage to potentially exposed non-target organisms, in that dsRNA is a natural molecule that is rapidly degraded in soils, waterbodies and plants (Parker et al., 2019; Bachman et al., 2020). The elimination of applied dsRNA in the agroecosystem after the relevant period of pest management not only reduces non-target organisms’ exposure to dsRNA, but also limits the target’s duration of exposure (especially in the case of dsRNA spray), thereby reducing the chance of resistance development. Furthermore, limiting the period of exposure can encourage the persistence of specialized biocontrol agents that rely on host pest availability (Willow et al., 2021b).

## Uniting Conservation Biocontrol and RNAi in the UN Decade on Ecosystem Restoration

The UN Environment Programme (UNEP) and the Food and Agriculture Organization (FAO) of the UN have acknowledged the urgency to restore damaged ecosystems; and that the required restoration measures are fundamental to sustainability goals including security of food and water resources, biodiversity conservation, poverty eradication and combating climate change. UNEP and FAO recognize the role of numerous restoration initiatives already led by citizens, farmers, businesses and governments. They also recognize that rural- and especially indigenous communities have been models for ecosystem preservation, and that building on their knowledge is vital for success of this decade’s global initiative. However, new technologies will play a pivotal role in achieving sustainability goals, especially when considering the current momentum in both technological development and human population growth. It is our view that combining conservation biocontrol-enhancing techniques with the use of both transgene- and spray-based RNAi technologies (Figure 1) represents a potentially viable and ecologically sustainable IPM strategy achievable across a global representation of agroecosystems. A primary aim of ecosystem restoration in agricultural landscapes should be enhancing connectivity- and expansion of habitats that promote healthy populations of biocontrol agents present in local and regional species pools; a better understanding of the habitat requirements of different biocontrol agents is a vital step toward achieving this aim. Accompanying these measures with RNAi-based crop protection can reinforce pest management efficacy. This interdisciplinary approach can help prevent agrochemical damage to non-target organisms, promoting biodiversity conservation. Employing cropping systems such as trap cropping, mixed cropping (Rosado et al., 2021) and agroforestry (Jose, 2009; Sagastuy and Krause, 2019; Campera et al., 2021; Gama-Rodrigues et al., 2021) has further potential to promote biodiversity conservation. Moreover, agroecological practices that promote crop diversification are more likely to have a positive effect on food security (Bezner Kerr et al., 2021). The abovementioned cropping systems could achieve greater success in pest management, biodiversity conservation and food security when implemented alongside RNAi crop protection technology.

Of notable importance for biocontrol of many crop pests is the continuous presence and services provided by specialist natural enemies such as parasitoids. It is conceivable that large-scale use and high efficacy of an applied pesticide (e.g., dsRNA) can reduce pest (host) populations to levels insufficient for sustaining local and/or regional parasitoid populations. This can be combated by preserving and/or restoring refuge habitats for pest species and their co-evolved parasitoids, both in and around the focal agroecosystem, a tactic that would help to suppress development of resistance, be applicable to all crop‒pest systems, and conform to the UN’s ecosystem restoration initiative.

Given dsRNA’s sequence-specificity and the potential this holds for managing pathogens and animal pests without directly affecting non-target organisms, examining the benefits (e.g., economic, crop yield, biodiversity) of combining conservation biocontrol practices with RNAi technology represents promising and unexplored territory. Greenhouse studies could be the first step to study the combined, potentially synergistic efficacy of these- or similar IPM approaches; augmented biocontrol is already a common technique for regulating pests in greenhouses, and could act as a useful model for conservation biocontrol. Being limited to closed systems, greenhouse studies are well suited for disentangling complex interactions between techniques while avoiding potential confounds. Ultimately, greenhouse studies would guide subsequent investigations into the use of such strategies in practical field scenarios, and provide insight for the betterment of greenhouse- and vertical farm crop protection. Studies examining the benefits of combining conservation biocontrol practices with RNAi technology should be conducted with regard to various crop‒pest systems, in order to maximize scientific value in a global context.

IPM has been the focus of much research for decades, yet there remains a global insufficiency of strategies mobilized for ensuring sustainability in both crop productivity and agrobiodiversity conservation. For example, regulators such as the EU define IPM policies, yet do not adequately support IPM practice (Ortega-Ramos et al., 2022). This is a complex problem that likely involves many players, from landowners and farmers to large companies and governing bodies. Leading authorities in IPM science, such as the International Organisation for Biological Control (IOBC), should assume a key role in developing conservation biocontrol-based IPM solutions that utilize RNAi technology, and serve as ambassadors to both industries and governing bodies, to promote significant progress towards global food security and agrobiodiversity conservation.

RNAi technology is not a stand-alone solution to achieving global food security and agrobiodiversity conservation. However, adopting sufficient conservation biocontrol measures, and combining these techniques with RNAi technology, may significantly increase the success of sustainable crop protection, given the low environmental risk of RNAi products compared to conventional pesticides. Non-RNAi plant biotechnologies with narrow-spectrum activity (e.g. gene editing, cisgenesis, marker-assisted selection) represent additional techniques that may have roles in facilitating sustainable IPM alongside conservation biocontrol measures. These could also be examined with regard to compatibility with biocontrol-enhancing measures. The current push for reducing humanity’s ecological footprint through the UN’s global initiative aimed at restoring damaged ecosystems, together with the recent advancements in RNAi research and utility, calls for innovative solutions relating to the interdisciplinary perspective introduced here.

## Data Availability

The original contributions presented in the study are included in the article/Supplementary Material, further inquiries can be directed to the corresponding author.
